# Ultrasound in Pigmented Rice Processing: A Versatile Approach for Anthocyanin Retention and Extraction: A Review

**DOI:** 10.1002/fsn3.72476

**Published:** 2026-10-11

**Authors:** Jingxian An, Chong Liu, Kwan‐Mo You

**Affiliations:** ^1^ Department of Food Science Purdue University West Lafayette Indiana USA; ^2^ Department of Chemical and Materials Engineering The University of Auckland Auckland New Zealand; ^3^ Department of Food Science and Technology Pukyong National University Busan Republic of Korea

**Keywords:** anthocyanin retention, extraction optimization, pigmented rice, starch modification, ultrasound technology

## Abstract

Pigmented rice contains anthocyanins and other bioactive phenolic compounds; however, its processing is challenged by the relatively low starch digestibility of some pigmented varieties, prolonged drying times, anthocyanin degradation, and inefficient pigment recovery. This review evaluates ultrasound as a complementary technology for addressing these challenges, focusing on three major applications: starch modification, ultrasound‐assisted drying, and anthocyanin extraction. Specifically, ultrasound‐induced physical effects can disrupt starch‐granule organization, alter molecular order, and thereby modulate starch functionality and digestibility. When combined with enzymatic or hydrothermal treatments, ultrasound may further accelerate structural modification, although the resulting effects depend on the treatment conditions and the specific rice matrix. In drying applications, ultrasound enhances heat and mass transfer and shortens processing time, thereby limiting prolonged thermal exposure. Similarly, in extraction, ultrasound‐assisted methods promote solvent penetration, matrix disruption, and pigment diffusion, collectively improving the recovery of anthocyanins and other bioactive compounds from rice grains and milling byproducts. Nevertheless, these effects are strongly influenced by ultrasound frequency, power, treatment duration, temperature, reactor configuration, and solvent system, and excessive treatment may in turn promote pigment degradation. Overall, ultrasound represents a parameter‐dependent complementary approach for improving processing efficiency, anthocyanin recovery, and the functional quality of pigmented‐rice products, though direct evidence for anthocyanin preservation during ultrasound‐assisted rice processing remains comparatively limited.

## Graphical abstract

Ultrasound‐induced cavitation facilitates starch modification, moisture transfer, and anthocyanin release from pigmented rice. Its effectiveness depends strongly on processing parameters and matrix characteristics. Careful optimization is therefore required to improve processing efficiency and anthocyanin recovery while minimizing pigment degradation.

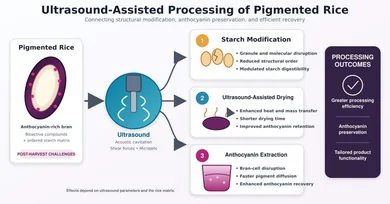

## 1 Introduction

Rice ( *Oryza sativa* L.) has sustained a large portion of the world's population over millennia. Archaeobotanical evidence from the lower Yangtze region indicates an early rice‐domestication process by approximately 9400 years ago (Zuo et al. 2017). Pigmented rice varieties, including black, red, purple, and purple‐red rice, are distinguished from non‐pigmented rice by their characteristic colors and their abundance of bioactive compounds, particularly flavonoids, anthocyanins, and proanthocyanidins. Among anthocyanins, cyanidin‐3‐glucoside is the predominant anthocyanin, reaching concentrations up to 470 mg/100 g, followed by peonidin‐3‐glucoside at up to 40 mg/100 g (Deng et al. 2013; Mapoung et al. 2023). However, these concentrations vary considerably among cultivars. For example, the concentrations of both anthocyanins in the Korean black rice varieties generally followed the order Heugnam (HN) > Heugjinju (HJJ) > Heugkwang (HK) (Kim et al. 2014), while cyanidin‐3‐glucoside concentrations were higher in dark‐purple rice grains than in medium‐purple grains (Rahman et al. 2013). The predominant phenolic compounds also differ among color types: whereas anthocyanins are particularly abundant in black and purple rice, the pigmentation of red rice is more strongly associated with proanthocyanidins, and other phenolic compounds concentrated in the outer layers of the caryopsis (Das and Bhattacharya 2019).

This distinctive composition has stimulated increasing consumer and industrial interest in pigmented rice. In several Asian countries, red and non‐glutinous black rice are consumed as cooked whole grains, whereas glutinous black and purple rice are commonly used in traditional desserts, snacks, and other processed foods (Samyor et al. 2017; Tananuwong and Tewaruth 2010). Nevertheless, it remains a specialty crop with more limited cultivation and commercial availability than conventional non‐pigmented rice. Grain yield varies considerably among cultivars, growing seasons, and water regimes because of differences in genotype, environmental conditions, and agronomic practices (Agustin et al. 2023; Jaksomsak et al. 2021). Moreover, continued dependence on traditional landraces, together with limitations in seed supply and production capacity, can constrain the ability of pigmented‐rice production to meet increasing market demand (Mbanjo et al. 2020).

Alongside these cultivation and supply constraints, the expanding utilization of pigmented rice presents several post‐harvest processing challenges. Its starch structure can limit digestibility and processing performance, whereas prolonged modification, cooking, and drying may promote anthocyanin degradation. Efficient recovery of anthocyanins from rice grains and processing byproducts presents an additional challenge. Addressing these interconnected challenges requires a processing technique capable of modifying starch structure, limiting unnecessary anthocyanins, and enabling efficient extraction. Ultrasound is particularly relevant in this context because it can modify starch structure, enhance heat and mass transfer, shorten processing time, and facilitate anthocyanin extraction.

Accordingly, this review examines ultrasound‐assisted post‐harvest processing of pigmented rice, with particular emphasis on starch modification, anthocyanin retention during drying, and anthocyanin extraction. The health significance and applications of pigmented‐rice anthocyanins are first discussed to establish why their preservation and recovery are important. Pre‐harvest production factors are briefly introduced only to provide context and are otherwise beyond the scope of this review.

To identify literature relevant to this focus, a search was conducted using Google Scholar, focusing on publications addressing pigmented rice processing and anthocyanin extraction. The search included peer‐reviewed journal articles published from 2010 onward, encompassing both original research and review papers written in English. Search terms included combinations of “pigmented rice,” “red rice,” “purple rice,” “black rice,” “anthocyanin,” “extraction,” “starch,” and “ultrasound.” Non‐peer‐reviewed sources, conference abstracts, and non‐English‐language publications were excluded from consideration.

## 2 The Role of Anthocyanins in Pigmented Rice: Applications

Anthocyanins contribute substantially to the antioxidant capacity of pigmented rice. In vitro and preclinical studies indicate that pigmented‐rice anthocyanins and anthocyanin‐rich extracts can modulate oxidative stress, inflammatory responses, glucose metabolism, and other biological pathways (Deng et al. 2013; Leonarski, Kuasnei, Cesca, et al. 2024; Mbanjo et al. 2020). These findings support further investigation of pigmented rice as a functional food ingredient, but they should not be interpreted as evidence that its consumption prevents or treats cardiovascular disease, diabetes, hypertension, cancer, or other chronic diseases in humans.

The nutritional and technological properties of pigmented rice support its use as a whole grain and as an ingredient in functional foods. In addition to anthocyanins, pigmented rice provides proteins, minerals, B vitamins, and other phytochemicals (Mbanjo et al. 2020; Samyor et al. 2017). For example, anthocyanin‐rich black‐rice extract has been incorporated into bread, where it increased antioxidant content and altered in vitro starch digestibility (Sui et al. 2016). Growing interest in naturally derived colorants and bioactive ingredients has also encouraged the development of pigmented‐rice products and the valorization of their processing byproducts (Samyor et al. 2017).

Beyond direct consumption and food fortification, the valorization of anthocyanin‐rich processing byproducts offers another important application pathway. Pigmented‐rice bran, a major byproduct of rice milling, is particularly rich in anthocyanins but has traditionally been used as animal feed or discarded as a low‐value material (Zhang et al. 2023). Its recovery and utilization can provide natural colorants and functional ingredients while improving the economic and environmental value of rice processing. The anatomical structure of black rice, the predominant localization of anthocyanins within the outer bran layers, and the potential applications of different grain fractions are illustrated in Figure 1.

**FIGURE 1:**
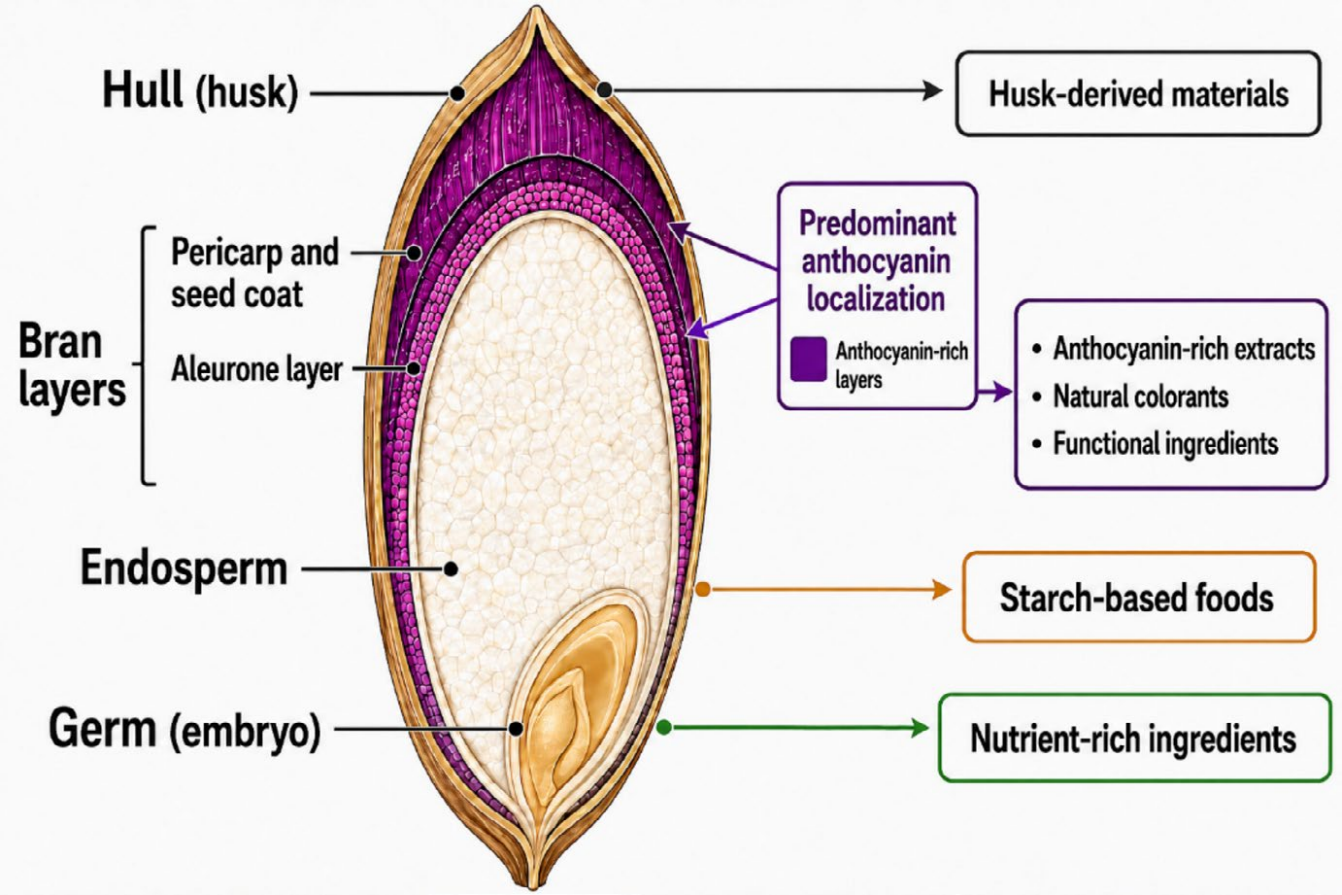
Schematic representation of the anatomical structure and potential applications of black rice grain fractions. Anthocyanins are predominantly localized in the outer bran layers, particularly the pericarp, seed coat, and aleurone layer.

Realizing these applications, however, depends on first recovering the anthocyanins from the bran matrix. Once recovered from these anthocyanin‐rich bran layers, the pigments can be utilized not only in functional foods but also in other value‐added applications. For example, pigmented‐rice anthocyanins have been incorporated into pectin/chitosan composite films as pH‐sensitive indicators for monitoring meat freshness (Zeng et al. 2023). More broadly, pigments extracted from rice and other cereals are attracting increasing interest because of consumer preference for natural colorants over synthetic alternatives (Shukla et al. 2022). These diverse applications highlight the importance of processing technologies that can preserve anthocyanins within pigmented‐rice products and efficiently recover them from rice grains and milling byproducts.

## 3 Processing Challenges of Pigmented Rice

Despite these advantages, pigmented rice presents several processing challenges. Firstly, non‐pigmented rice, in both purified starch and flour forms, demonstrates higher starch digestibility compared to pigmented rice varieties (Almeida et al. 2019; Ratseewo et al. 2019). This lower digestibility is attributed to endogenous anthocyanins, which can inhibit starch digestion (Ni et al. 2024). Secondly, processing pigmented rice typically requires longer cooking times than non‐pigmented varieties. For example, in the production of glutinous rice products, such as frozen sweet dumplings, the extended soaking and heating periods can result in substantial anthocyanin loss (Wang et al. 2024).

Among these challenges, anthocyanin degradation during prolonged processing is a major concern. Extended heat treatment can degrade monomeric anthocyanins, resulting in reduced pigment content and color intensity (Devi and Badwaik 2022). Similarly, prolonged thermal drying may further reduce the retention of these heat‐sensitive compounds. Efficient pigment extraction presents an additional difficulty because anthocyanins must be released from the outer bran layers and transferred into the extraction solvent, while prolonged exposure to oxygen and heat during extraction may promote their oxidation and degradation. Thus, both anthocyanin preservation and efficient recovery depend strongly on minimizing unnecessary processing time and intensity.

These challenges are closely interconnected with the starch properties of pigmented rice. Its relatively ordered starch‐granule structure, together with interactions between starch and endogenous phenolic compounds, can restrict enzyme accessibility and require longer soaking, cooking, or structural‐modification treatments. However, increasing treatment duration or intensity to improve starch functionality may simultaneously accelerate anthocyanin degradation. Likewise, extending extraction may enhance pigment release but also increase oxidative losses. An appropriate processing technology must therefore balance three related objectives: effective starch modification, shorter cooking and drying times, and rapid anthocyanin recovery with minimal pigment degradation.

It is worth noting that anthocyanin degradation can occur throughout multiple stages of processing and subsequent food preparation. Anthocyanin stability is influenced by processing conditions and food‐matrix characteristics, although the magnitude of these effects is highly matrix‐dependent (Chen et al. 2023). In pigmented‐rice applications, the final food matrix may likewise affect anthocyanin functionality and stability; for example, anthocyanin‐rich black‐rice extract has been incorporated into bread (Sui et al. 2016). Therefore, observations obtained from non‐rice matrices should be interpreted as mechanistic support rather than direct evidence for pigmented rice. This underscores the importance of minimizing unnecessary processing severity throughout the processing chain. With this in mind, the following sections evaluate ultrasound as a potential complementary approach for starch modification, processing‐time reduction, and anthocyanin extraction.

## 4 Rationale for Ultrasound Technology in Addressing Pigmented Rice Challenges

Ultrasound technology used in food processing can be broadly categorized into two frequency ranges based on their applications. High‐frequency ultrasound (100 kHz–1 MHz), typically operating at intensities below 1 W/cm^2^, is primarily used for non‐destructive analytical purposes, such as monitoring food composition and quality. In contrast, low‐frequency, high‐intensity ultrasound (16–100 kHz) is widely applied in food processing to induce physical or chemical modifications, or to extract target metabolites; depending on the structural properties of the food matrix, such treatment may or may not compromise the integrity of these compounds (Soria and Villamiel 2010; São José et al. 2014). Given its capacity to induce these targeted physical and chemical modifications, low‐frequency ultrasound is particularly relevant to the processing challenges addressed in this review—namely, starch modification, drying, and anthocyanin extraction in pigmented rice.

The effects of low‐frequency ultrasound depend on the processing medium and arise mainly from physical and sonochemical mechanisms. In liquids, alternating compression and rarefaction waves induce acoustic cavitation, and the subsequent collapse of cavitation bubbles generates shear forces, microjets, and localized pressure and temperature gradients (Kerboua et al. 2022; Kentish and Feng 2014). In starch–water systems, these physical effects can disrupt starch‐granule organization (Keeratiburana et al. 2020). During anthocyanin extraction and liquid‐phase drying pretreatment, they can also enhance solvent penetration and pigment diffusion and increase tissue porosity (Kerboua et al. 2022). In contact or airborne ultrasound‐assisted drying, moisture migration is instead promoted mainly through mechanical vibration, the repeated compression–expansion or “sponge effect,” and disruption of the boundary layer (Kentish and Feng 2014). Under excessive treatment conditions, ultrasound may also generate reactive species, potentially causing molecular modification or anthocyanin degradation (Kentish and Feng 2014).

Notably, this technology is mature rather than novel, with research on ultrasonic depolymerization dating back to 1933. Sonication has long been recognized as an efficient route for depolymerization, offering improved reaction rates, yields, and chemo‐, regio‐, and stereoselectivity, typically achieved using simple immersion probes operating at 20 kHz (Mason et al. 2011). Despite this well‐established mechanistic foundation, its application to pigmented rice processing remains comparatively underexplored—and it is precisely this targeted application, rather than the underlying technology itself, that constitutes the novelty of the present review.

### 4.1 Effective Starch Modification Properties

Ultrasound is an established physical method for starch modification. In aqueous systems, cavitation‐induced shear and microjets can produce surface erosion, cracks, depressions, and increased porosity in starch granules, although the resulting morphology depends on starch composition and treatment severity (Bonto et al. 2021; Yang et al. 2019). Starches with different granule sizes and amylose contents can therefore respond differently to otherwise similar ultrasound treatments (Hu et al. 2019).

When applied to rice starch, ultrasonic treatment can alter granule morphology and molecular organization, thereby enhancing swelling properties, reducing short‐range molecular order and relative crystallinity, and modifying gelatinization characteristics (Wang et al. 2024). These changes arise mainly from acoustic cavitation, which generates intense shear forces, localized pressure and temperature gradients, and reactive species in starch–water systems (Keeratiburana et al. 2020). However, the extent and direction of these modifications depend on ultrasound intensity, treatment duration, temperature, and starch composition. Representative rice‐specific studies and their reported structural and functional modifications are summarized in Table 1. Collectively, these studies demonstrate that ultrasound can modify rice starch at multiple structural levels, but the resulting functionality is strongly dependent on the rice starch type and treatment conditions.

**TABLE 1:** Representative studies on ultrasound‐induced modifications of rice starch.

Rice material	Treatment conditions	Main structural modifications	Functional effects	References
Rice starch	150–600 W, 25°C, 20 min	Surface fissures and pores; limited changes in fine molecular structure; A‐type crystalline pattern retained	Increased peak and breakdown viscosities; decreased pasting temperature, peak time, and gelatinization enthalpy	Yang et al. (2019)
Rice starch	Vacuum at 0.09 MPa combined with ultrasound at 300 W	Shorter molecular chains; reduced lamellar order and relative crystallinity	Reduced gelatinization temperature and enthalpy; altered pasting behavior	Li et al. (2022)
Granular cold‐water‐swelling rice starch	Ultrasound‐assisted alcoholic–alkaline treatment at different ultrasound power levels	Disruption and reorganization of granular structure	Improved cold‐water swelling and solubility and modified pasting properties	Kunyanee et al. (2023)
Purple‐rice starch	Heat–moisture treatment at 110°C for 4 h combined with ultrasound for 30 min	Rougher granule surfaces; altered short‐range order; decreased relative crystallinity; A‐type pattern retained	Enhanced paste fluidity and resistance to deformation	Su et al. (2024)

### 4.2 Effective Processing Time Reduction

Ultrasonication represents a versatile non‐thermal technology widely adopted in food processing applications. In rice systems, ultrasound has been successfully integrated with other processing techniques, including enzymatic hydrolysis, to increase structural accessibility and improve processing efficiency (Keeratiburana et al. 2020). However, the magnitude of these effects depends strongly on the food matrix, ultrasound conditions, and the accompanying processing method. Therefore, reductions in processing time and improvements in product quality observed in non‐rice food systems should not be directly extrapolated to pigmented rice. In the following sections, rice‐specific evidence is emphasized where available, while findings from other plant matrices are used primarily to provide mechanistic support for the potential application of ultrasound in starch modification, drying, and anthocyanin extraction.

The following Sections 5–7 will examine in detail the application of ultrasound technology to pigmented rice processing, specifically addressing three key aspects: starch modification to modulate digestibility and its potential implications for anthocyanin retention, prevention of anthocyanin loss during drying processes, and optimization of anthocyanin extraction efficiency.

## 5 Ultrasound‐Assisted Starch Modification: Modulating Digestibility and Potential Implications for Anthocyanin Retention

### 5.1 Modulation of Starch Digestibility

Beyond traditional methods such as annealing and heat–moisture treatment, several mechanically assisted approaches have been developed for starch modification. Although pulsed electric field (PEF) and microwave treatments can be applied to starch modification and bioactive‐compound extraction from pigmented rice (Salee et al. 2023), these treatments may increase slowly digestible starch or resistant starch, making them less suitable when enhanced starch digestibility is desired (Bai et al. 2021; Zeng et al. 2016; Wu et al. 2019; Chi et al. 2023). In contrast, ultrasound can disrupt starch‐granule organization, reduce molecular order, and increase enzyme accessibility, while facilitating the release of anthocyanins and other bioactive compounds from the rice matrix (Yusoff et al. 2022; Su et al. 2024). However, this improvement may be partially offset by starch retrogradation during subsequent cooling and storage, as reassociation of amylose and amylopectin can rebuild ordered structures and restrict enzyme access. Such retrogradation is also functionally important: moderate molecular reassociation may strengthen rice‐noodle structure, whereas excessive retrogradation may cause hardening and syneresis in cooked or frozen rice products (Yu et al. 2013; Bonto et al. 2021; Geng et al. 2021). Therefore, the effects of ultrasound should be evaluated by balancing starch digestibility, anthocyanin bioaccessibility, and the quality requirements of the final product.

### 5.2 Combination With Other Techniques to Preserve Anthocyanins

A key advantage of ultrasound is its flexibility in combination with other processing techniques, which can facilitate starch modification and improve processing efficiency in rice systems (Kunyanee et al. 2023; Keeratiburana et al. 2020). In particular, ultrasound‐assisted enzymatic treatment can increase starch surface accessibility and facilitate enzyme–substrate interactions, thereby accelerating structural modification. For example, Geng et al. (2021) reported that ultrasound‐assisted cellulase treatment improved the textural properties and modified the in vitro starch digestibility of brown rice noodles, while Lu et al. (2023) demonstrated improved freeze–thaw stability of brown rice following ultrasound‐assisted cellulase pretreatment. Zhang et al. (2015) likewise showed that ultrasonic enzyme treatment improved the cooking and eating quality of brown rice. Collectively, these studies demonstrate that ultrasound can enhance the efficiency and functional outcomes of enzymatic processing in rice systems. Although shorter or milder processing may reduce the thermal exposure of anthocyanins, this potential preservation benefit should be distinguished from direct evidence of anthocyanin retention.

Evidence directly linking shortened ultrasound‐assisted processing to improved anthocyanin retention in pigmented rice remains limited and condition‐dependent. Ultrasound combined with hydrothermal treatment has, for instance, been reported to alter brown‐rice cooking quality and processing behavior (Liu et al. 2024), while ultrasonication combined with germination and steaming has been shown to change the starch, phenolic, and anthocyanin characteristics of black rice (Athoi et al. 2024). Similarly, ultrasonic‐assisted immersion has been investigated as part of black‐rice parboiling workflows (Wu et al. 2023). Together, these studies demonstrate that ultrasound can modify processing performance and bioactive‐compound profiles, but they do not establish a general preservation effect. Anthocyanin retention must therefore be evaluated directly for each rice cultivar, process sequence, and ultrasound condition.

## 6 Potential of Ultrasound‐Assisted Drying to Limit Anthocyanin Degradation in Pigmented Rice

After harvesting, paddy rice must be dried to a moisture level suitable for safe storage and milling. Drying conditions, in turn, can substantially affect phenolic compounds, anthocyanins, and antioxidant activity in pigmented rice (Ratseewo et al. 2020). In particular, higher drying temperatures and prolonged storage can accelerate the loss of heat‐sensitive phenolic compounds; consequently, drying temperature and time must be selected for the specific cultivar and target quality attributes rather than applying a universal threshold (Lang et al. 2019; Ramos et al. 2022). Against this backdrop, ultrasound‐assisted drying is of particular interest, since structural disruption and enhanced moisture migration may shorten drying exposure—though direct evidence demonstrating superior anthocyanin retention in pigmented rice remains limited.

### 6.1 Potential Retention of Bioactive Compounds

Rather than being used alone, ultrasonic irradiation is typically combined with other techniques such as infrared or microwave drying to achieve effective drying while maintaining food appearance and bioactive compounds. Evidence from non‐rice plant matrices suggests that ultrasound pretreatment may contribute to quality preservation by reducing the activity of enzymes such as polyphenol oxidase and peroxidase, which are associated with color deterioration and bioactive compound loss (Kutlu et al. 2022; Xu et al. 2022). Ultrasound treatment has also been reported to help maintain color intensity and visual appearance in several food matrices (Kutlu et al. 2022). For example, Baeghbali et al. (2020) found that ultrasound pretreatment combined with infrared drying effectively preserved the color of dried okra. Although direct evidence in pigmented rice remains limited, these findings from other plant matrices provide a useful mechanistic rationale for exploring ultrasound‐assisted drying as a potential strategy for improving color and bioactive‐compound retention in pigmented rice.

### 6.2 Drying Time Reduction

Ultrasound pretreatment can alter plant‐tissue structure and increase porosity, thereby facilitating the migration of free, immobilized, and some bound water (Guo et al. 2020; Santos et al. 2024). Evidence for these mechanisms has, however, been demonstrated primarily in non‐rice plant matrices, where ultrasound has been associated with enhanced heat and mass transfer and shorter drying times. For example, ultrasound‐assisted drying reduced drying time in banana and saffron systems, although the magnitude of the effect depended on product structure and pretreatment conditions (Llavata et al. 2020; Pei et al. 2021). Similarly, ultrasound pretreatment combined with far‐infrared drying has accelerated moisture removal in other agricultural products (Shang et al. 2023). Nevertheless, these quantitative effects should not be directly extrapolated to pigmented rice, since tissue structure, composition, geometry, and acoustic coupling differ among materials.

Given this gap, rice‐specific and ultrasound‐specific evidence must be distinguished. Far‐infrared drying has been evaluated in pigmented rice and can affect starch digestibility, phenolic compounds, and anthocyanins, but the rice studies cited here did not incorporate ultrasound (Ratseewo et al. 2020, 2022). Conversely, ultrasound‐assisted far‐infrared drying has shortened drying time or improved selected quality attributes in non‐rice matrices such as potato (Xi et al. 2020). Ultrasound combined with microwave treatment has likewise been reported to alter rice drying behavior, color, texture, and other quality attributes (Zhang et al. 2024), although this does not by itself demonstrate anthocyanin preservation in pigmented rice. Taken together, ultrasound‐assisted infrared drying is a plausible strategy, but direct pigmented‐rice studies measuring anthocyanin retention are required before a preservation advantage can be concluded.

## 7 Ultrasound‐Enhanced Anthocyanin Extraction: Optimization and Efficiency

### 7.1 Potential Economic Advantages of Ultrasound‐Assisted Extraction

While anthocyanins and other phenolic compounds have been extensively studied in fruit sources such as blueberries and blackberries, pigmented rice represents a comparatively underexploited and cost‐effective source (Colombo et al. 2023). This is underscored by the scale of pigmented rice production and its associated byproducts: pigmented rice varieties such as black, red, and purple rice are widely cultivated across Asian countries, including China, Japan, India, Indonesia, and Thailand (Itani and Ogawa 2004; Kammapana 2023; Kushwaha et al. 2020), and their processing generates substantial quantities of rice bran and husk byproducts that remain largely underutilized or discarded (Tan et al. 2023). Given this abundant feedstock availability, ultrasound‐assisted extraction offers a potentially scalable and cost‐effective route for valorizing these byproducts into anthocyanin‐rich functional ingredients—an advantage not readily available when relying on conventional fruit‐derived sources or limited whole‐grain material.

The feasibility of large‐scale anthocyanin recovery depends on extraction efficiency, solvent use, energy input, and equipment configuration. In this context, UAE and microwave‐assisted extraction (MAE) can shorten extraction time relative to prolonged conventional solvent treatments, but their performance remains matrix‐ and parameter‐dependent (Leonarski, Kuasnei, dos Santos, et al. 2024). Notably, Leonarski, Kuasnei, dos Santos, et al. (2024) evaluated UAE and MAE as separate approaches for black‐rice bran, whereas Jha et al. (2017) optimized a sequential, combined UAE–MAE process for black‐rice husk; these distinct process configurations should therefore not be conflated. Taken together, available studies support the technical potential of assisted extraction, but claims of lower cost, scalability, or superior biological activity require process‐specific techno‐economic analysis and, for health outcomes, evidence beyond compositional and in vitro assays.

### 7.2 Compatibility With Bioactive Compounds

Ultrasound‐assisted extraction enhances the recovery of bioactive compounds through multiple mechanisms, including acoustic cavitation, shear forces, microjet formation, localized pressure, agitation and radical formation (Colombo et al. 2023). Among these, acoustic cavitation is considered the primary physical driver: it can only occur in a liquid medium (e.g., water or aqueous ethanol), where the formulation and violent collapse of microbubbles disrupt plant cell walls and membranes, thereby facilitating the release of anthocyanins into the solvent (Kerboua et al. 2022). This mechanism is illustrated in Figure 2, which depicts the disruption of the pigmented rice bran matrix under ultrasonic cavitation and the subsequent release of anthocyanins into the extraction solvent.

**FIGURE 2:**
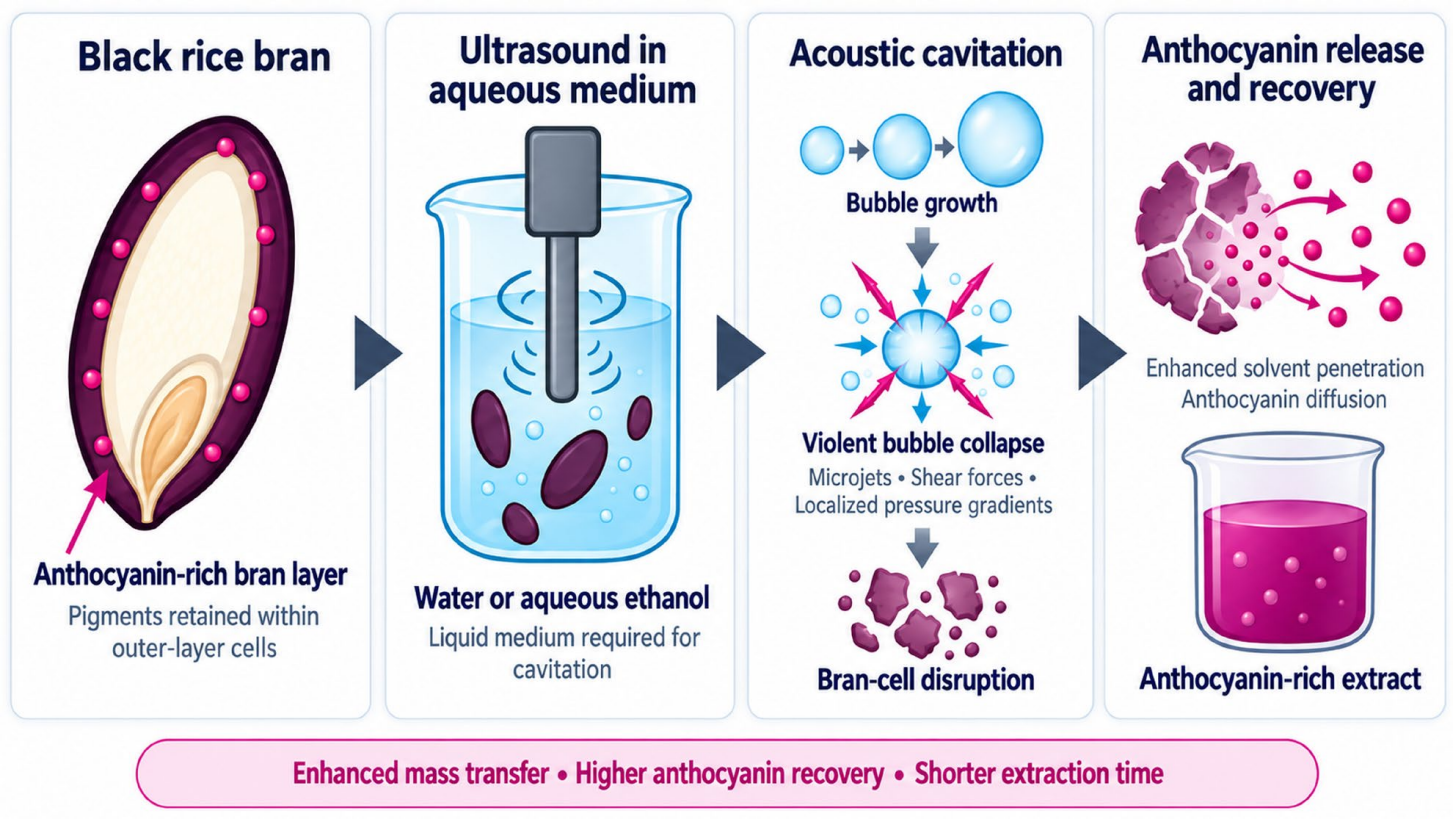
Schematic illustration of ultrasound‐assisted extraction of anthocyanins from black rice. Acoustic cavitation generates microbubbles, shear forces, and microjets that disrupt the anthocyanin‐rich bran layer and facilitate the release and diffusion of anthocyanins into the surrounding solvent.

The magnitude of ultrasound‐assisted extraction is not uniform; it varies with frequency, power or acoustic intensity, treatment time, temperature, reactor geometry, and the physicochemical properties of the solvent and sample matrix (Engmann et al. 2015; Fu et al. 2021; Golmohamadi et al. 2013). Solvent composition, pH, and temperature also influence both anthocyanin solubility and stability (Win et al. 2024). Acidic conditions generally improve anthocyanin stability, whereas high temperatures and prolonged exposure accelerate degradation (Das et al. 2020). An extraction temperature of 60°C was optimal under the specific aqueous UAE conditions evaluated for black‐rice bran by Klaykruayat et al. (2025), but this value should not be treated as a universal optimum. Representative studies and their directly reported conditions and analytical responses are summarized in Table 2.

**TABLE 2:** Usage of ultrasound‐assisted extraction for pigmented rice.

Source material (references)	Solvent system	Extraction conditions	Key findings
Black glutinous rice (Pariyarath et al. 2018)	70% aqueous ethanol	UAE amplitude: 65% Extraction time: 5 min	Maximum yield: ○ Total phenolic content: 136.17 mg GAE/g The reported TAC unit was dimensionally ambiguous and is therefore not reproduced
Black rice husk (Jha et al. 2017)	67.34% aqueous ethanol	Sequential combined UAE‐MAE Sonication time: 10.02 min Sonication temperature: 49.46°C Liquid‐to‐solvent ratio: 40.79 mL/g Microwave time: 31.11 s	Experimental values under the optimal conditions: ○ TPC: 1.72 mg GAE/g ○ Anthocyanins: 3.36 mg/100 g ○ Flavonoids: 3.01 mg/100 g ○ Antioxidant activity: 100%
Black rice bran (Klaykruayat et al. 2025)	Water	Ultrasonic bath frequency: 35 kHz Temperature: 60°C Time: 30 min Liquid‐to‐solid ratio: 21 mL/g	Observed total anthocyanin concentration: around 173.25 mg C3G equivalents/L
Black rice bran (Leonarski, Kuasnei, dos Santos, et al. 2024)	0.1 M citric acid‐ethanol, 60:40 (v/v)	UAE evaluated separately from MAE Temperature: 50°C Ultrasonic power: 380 W	UAE recovered 83.73% of the total anthocyanins, corresponding to 2.44 mg C3G/g. UAE and MAE were separate extraction approaches, not one combined process
Pigmented rice bran (Bunmusik et al. 2023)	60% aqueous methanol containing 0.1% citric acid	Ultrasonic bath frequency: 37 kHz Temperature: 30°C Time: 30 min Liquid‐to‐solid ratio: 20 mL/g	Yields ○ TAC: 3.15 mg C3G/g DW ○ TPC: 16.13 mg GAE/g DW ○ TFC: 4.04 mg QE/g DW
Defatted Vietnamese purple rice bran (Loan et al. 2024)	Acetate buffer (pH 3.0) containing 1% cellulase	Ultrasound‐assisted enzymatic extraction Liquid‐to‐solid ratio: 10 mL/g Temperature: 40°C Ultrasonic‐bath rated power: 110 W Instrument power setting: 40% Extraction approached equilibrium after approximately 60 min	Maximum TPC reported in the text: 60.89 mg GAE/g rice bran Fitted equilibrium value reported in the text: 60.98 mg GAE/g
Bran from four Thai rice cultivars (Peanparkdee et al. 2019)	65%–67% aqueous ethanol	Optimized UAE range: 51°C–54°C Optimized UAE time: 40–45 min	Aqueous ethanol at 65% (v/v) produced the highest overall phenolic‐acid, flavonoid, anthocyanin, TPC, DPPH, and FRAP responses The appropriate UAE range was 51°C–54°C and 40–45 min
Red rice bran (Wang et al. 2020)	Acidified 78.37% aqueous ethanol (pH 2.31)	Liquid‐to‐solid ratio: 17.46 mL/g Ultrasound power: 400 W Ultrasound frequency: 24 kHz Ultrasonication time: 55.23 min	Predicted anthocyanin yield: 5.799 mg/g Experimentally validated yield after rounded settings: 5.512 mg/g
Riceberry bran (Peanparkdee et al. 2018)	Ethanol or d‐limonene, evaluated separately	Ultrasonic‐bath frequency: 43 kHz Ethanol UAE: approximately 51°C, 40 min, and liquid‐to‐solid ratio 20 mL/g. d‐Limonene UAE: 60°C, 60 min, and liquid‐to‐solid ratio 5 mL/g	Ethanolic UAE produced the highest TPC, TFC, TAC, DPPH, and FRAP responses among the extraction‐method and solvent combinations evaluated.
Thai pigmented rice bran (Ratanasongtham et al. 2024)	Deep eutectic solvent: choline chloride‐ethylene glycol, 1:2 M ratio, containing 20% water	Ultrasound frequency: 37 kHz Temperature: 50°C Time: 40 min Liquid‐to‐solid ratio: 6 mL/g	Yields ○ TAC: 4.55 ± 0.09 mg C3G/g DW ○ TPC: 26.49 ± 0.62 mg GAE/g DW ○ TFC: 6.57 ± 0.55 mg QE/g DW DPPH inhibition: 77.83% ± 1.51%

*Note:* Ratios are expressed as solvent volume per unit solid mass where the original data permitted. Reported responses should not be compared directly across studies because sample bases, analytical standards, extraction configurations, and reporting units differed. For Pariyarath et al. (2018), the numerical TAC value was not reproduced because the unit reported in the source was dimensionally ambiguous.

Abbreviations: AAE, ascorbic acid equivalents; C3G, cyanidin‐3‐glucoside; DW, dry weight; GAE, gallic acid equivalents; MAE, microwave‐assisted extraction; QE, quercetin equivalents; TAC, total anthocyanin content; TFC, total flavonoid content; TPC, total phenolic content; UAE, ultrasound‐assisted extraction.

### 7.3 Stabilization of Anthocyanins Following Extraction

Following extraction, anthocyanins become susceptible to degradation caused by pH, temperature, light, and oxygen, potentially resulting in color loss and reduced antioxidant activity (Enaru et al. 2021). Maintaining acidic conditions and limiting exposure to heat, light, and oxygen are therefore fundamental for preserving anthocyanin‐rich extracts. Copigmentation or complexation with phenolic compounds, proteins, polysaccharides, and hydrocolloids can further improve stability by protecting the anthocyanin chromophore from degradation (Raharjo et al. 2019; Zhao et al. 2020).

When these measures alone provide insufficient protection during processing or prolonged storage, encapsulation offers a more robust stabilization strategy. Spray drying, freeze drying, and biopolymer‐based delivery systems can protect anthocyanins against adverse environmental conditions while facilitating their incorporation into food products. Studies on black rice anthocyanins have demonstrated effective encapsulation using maltodextrin, milk proteins, peptides, and other biopolymer wall materials (Aprodu et al. 2019; Norkaew et al. 2019). Thus, coupling ultrasound‐assisted extraction with appropriate post‐extraction stabilization is important for retaining the color, antioxidant activity, and functional value of anthocyanins recovered from pigmented rice.

## 8 Limitations and Scale‐Up Considerations

Despite these advantages, ultrasound‐assisted processing has several limitations. Its effects are highly dependent on frequency, power density, treatment duration, temperature, reactor configuration, and the physicochemical properties of the rice matrix, making direct comparison and process standardization difficult. Excessive sonication may generate localized heating and reactive radicals, potentially degrading anthocyanins and other phenolic compounds rather than preserving them. Moreover, non‐uniform acoustic fields and attenuation of ultrasonic energy in dense or heterogeneous materials can result in uneven treatment. At the industrial scale, equipment cost, probe erosion, energy efficiency, and reproducibility also require further evaluation. Therefore, ultrasound should not be regarded as universally superior to conventional processing; its benefits depend on careful parameter optimization and its appropriate integration with drying, enzymatic treatment, or extraction systems (Kentish and Feng 2014; Chavan et al. 2022; Shen et al. 2023).

## 9 Conclusion

Ultrasound is a versatile, parameter‐dependent tool for pigmented‐rice processing. In liquid systems, acoustic cavitation generates shear forces, microjets, and localized pressure and temperature gradients, which can modify starch structures and promote the release of bioactive compounds. These effects depend on frequency, power or acoustic intensity, duration, temperature, reactor configuration, solvent, and rice matrix. Among the applications reviewed, the strongest rice‐specific evidence currently concerns starch modification and ultrasound‐assisted extraction; by contrast, evidence that ultrasound consistently preserves anthocyanins during starch processing or drying is less direct and should be interpreted cautiously. Ultrasound can also be integrated with enzymatic, hydrothermal, microwave, or drying processes, but combined and sequential processes must be distinguished from stand‐alone UAE, as their effects are not necessarily additive or interchangeable. Beyond these mechanistic and evidentiary considerations, industrial implementation will further require reproducible acoustic‐field characterization, energy and solvent balances, equipment‐specific scale‐up, and techno‐economic evaluation. Ultrasound should therefore be considered a complementary processing technology rather than a universally superior replacement for conventional methods.

Future research directions should focus on:

1. Optimizing processing parameters (e.g., frequency, intensity, and duration) tailored to specific rice varieties and target compounds;
2. Investigating novel ultrasound‐assisted combinations with emerging technologies, such as pulsed electric fields, high‐pressure processing, or cold plasma;
3. Developing standardized protocols for different pigmented rice varieties to ensure reproducibility and quality control;
4. Scaling up ultrasound applications for industrial production while maintaining energy efficiency and product integrity;
5. Exploring the integration of ultrasound with green solvents (e.g., deep eutectic solvents or natural eutectic mixtures) to promote environmentally sustainable extraction processes;
6. Advancing techniques for the selective extraction of specific anthocyanin derivatives, such as cyanidin‐3‐glucoside, to support targeted functional food and nutraceutical applications; and
7. Conducting techno‐economic assessments and life cycle analyses to evaluate the long‐term feasibility of ultrasound‐assisted processing in commercial settings.

## Author Contributions

**Jingxian An:** conception, methodology, writing – original draft, data curation, software. **Chong Liu:** writing – review and editing. **Kwan‐Mo You:** writing – review and editing.

## Funding

The authors have nothing to report.

## Disclosure

*Author Responsibility Statement*: All authors have read and approved the final version of the manuscript. Jingxian An had full access to all of the data in this study and takes complete responsibility for the integrity of the data and the accuracy of the data analysis.

## Ethics Statement

This study does not involve any human or animal testing.

## Consent

The authors have nothing to report.

## Conflicts of Interest

The authors declare no conflicts of interest.

## Data Availability

Data sharing was not applicable to this article as no datasets were generated or analyzed during the current study.
